# Another Source of Near-Fatal Bleeding—Late-Onset Hemothorax Six Weeks After Uneventful Minimally Invasive Repair of Pectus Excavatum

**DOI:** 10.3390/children13081093

**Published:** 2026-08-17

**Authors:** Frank-Martin Haecker, Kai-Uwe Kleitsch

**Affiliations:** 1 Chest Wall Unit, Departement of Pediatric Surgery, Children’s Hospital of Eastern Switzerland, 9006 St. Gallen, Switzerland; kai-uwe.kleitsch@kispisg.ch; 2 Faculty of Medicine, University of Basel, 4001 Basel, Switzerland

**Keywords:** pectus excavatum, minimally invasive repair, late-onset hemothorax, accidental lesion of the internal mammary artery

## Abstract

**Highlights:**

Pectus surgeons are aware of the risk of cardiac perforation and/or accidental lesion of the internal mammary artery (IMA) during minimally invasive repair of pectus excavatum (MIRPE). However, delayed hemothorax six weeks after MIRPE is an uncommon and clin-ically important complication. We report on a 14-year-old adolescent who presented six weeks postoperatively after uneventful MIRPE with shortness of breath and dizziness. Presumed bleeding of the IMA was identified as the source of bleeding, and finally, con-servative management was successful.

**What are the main findings?**

- Not only during MIRPE, but also weeks after MIRPE, major complications may happen.
- Presumed bleeding of the IMA may be another source of near-fatal bleeding after MIRPE.

**What is the implication of the main finding?**

- Pectus surgeons have to be aware of this rare but serious complication after MIRPE.
- In selected cases, conservative management/watchful waiting may be successful even in patients with suspected major bleeding.

**Abstract:**

**Background/Objectives**: The minimally invasive repair of pectus excavatum (MIRPE) and its modifications represent the gold standard for surgical repair of chest wall deformities. Severe complications during or after MIRPE are rare but likely underreported in the literature. **Methods/Results**: We report the case of a 14-year-old adolescent who underwent an uneventful repair of an asymmetric pectus excavatum. Six weeks postoperatively, the patient presented with shortness of breath and dizziness. Clinical examination and imaging revealed a spontaneous massive right-sided hemothorax. Following chest tube placement, an initial 600 mL of bloody pleural fluid was drained. CT angiography demonstrated no active bleeding but raised suspicion of an injury to the right-sided internal mammary artery. After a total drainage of 1600 mL, the patient remained hemodynamically stable, and the chest tube was removed within 24 h. During 32 months of follow-up, the further clinical course was uneventful, and imaging demonstrated no secondary displacement of the implants. **Discussion/Conclusions**: Accidental injury to the internal mammary or intercostal vessels during pectus surgery is a rare but potentially life-threatening complication. Only a few cases of delayed hemorrhage following MIRPE have been reported, and in many a clear source of bleeding could not be identified. Pectus surgeons should be aware of this rare complication and should report such cases to improve understanding of its incidence and management.

## 1. Introduction

Since its introduction by Donald Nuss in 1998, the minimally invasive repair of pectus excavatum (MIRPE) has become the worldwide gold standard surgical technique for correcting congenital and acquired chest wall deformities in pediatric, adolescent, and adult pectus patients [1,2,3,4]. Over the past two decades, numerous technical modifications have been introduced to improve procedural safety, minimize complications, and optimize clinical outcomes [5]. Severe complications during and/or after MIRPE are rare but also underreported in the literature [6]. The most feared complication of MIRPE is cardiac perforation, followed by severe hemorrhage resulting from injury to the internal mammary artery (IMA). These complications have been reported both during bar placement and during bar removal (PBR) [1,7,8]. Vascular complications are very rare, with the occurrence of delayed bleeding events exceptionally uncommon. Pectus surgeons have to be aware of these complications which may happen not only during surgical repair and directly after surgery during hospital stay, but also after considerable delay. With our case report, we would like to raise awareness for a rare complication with late-onset hemothorax six weeks after MIRPE.

## 2. Case Report—Clinical Summary

**Figure 1:**
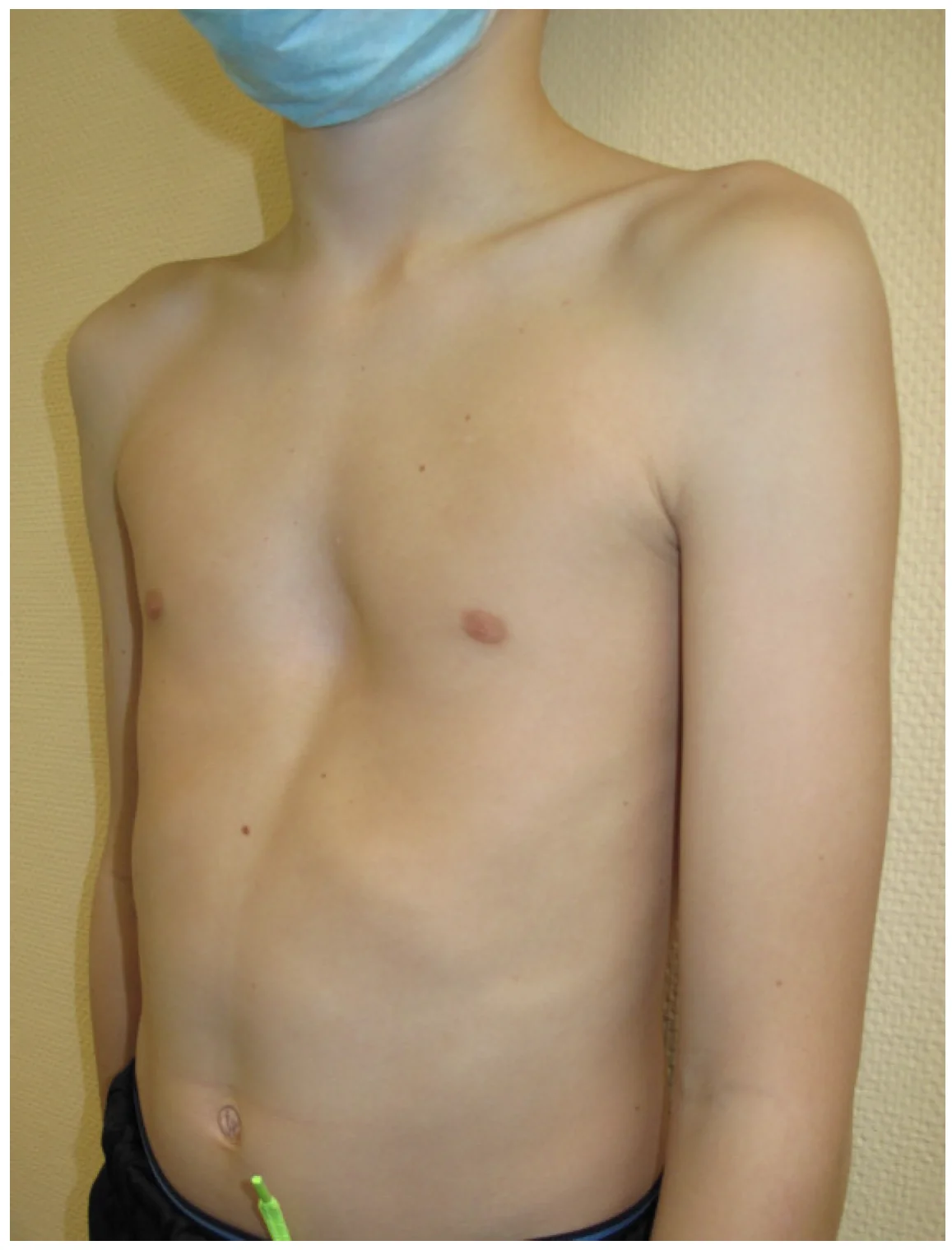
14-year-old patient with pectus excavatum before MIRPE.

**Figure 2:**
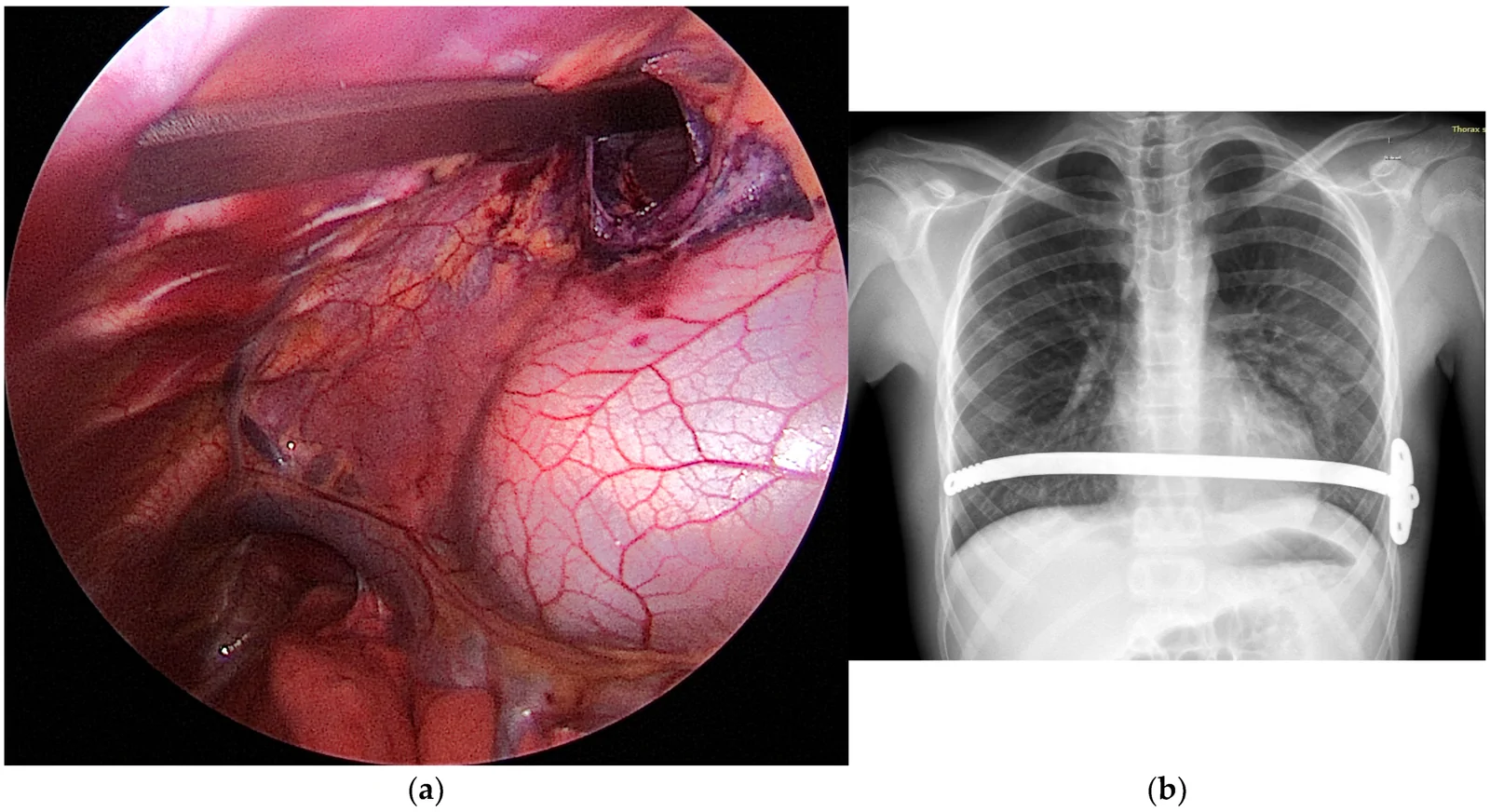
(**a**) Thoracoscopic view during MIRPE. (**b**) Chest x-ray after MIRPE.

**Figure 3:**
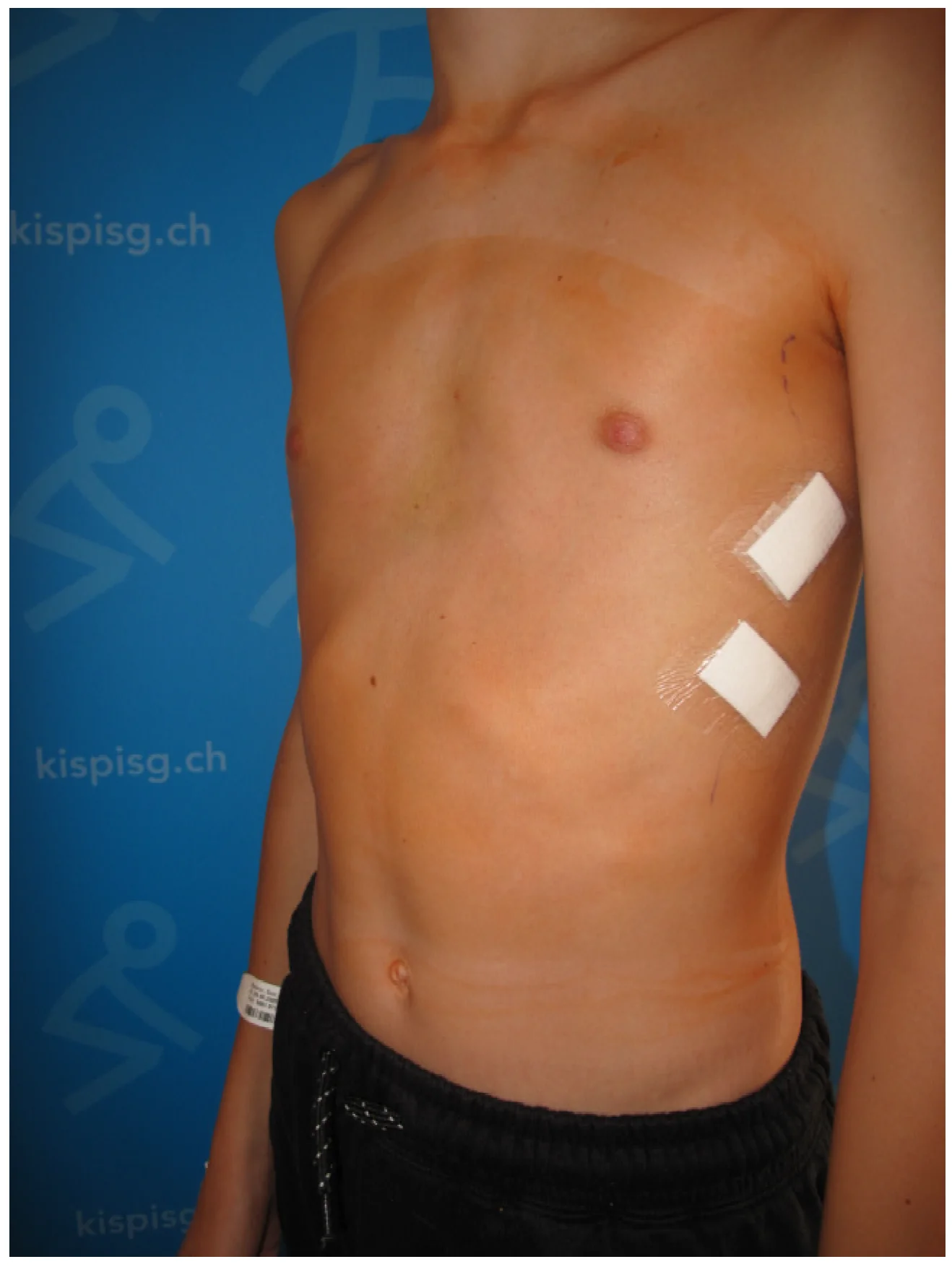
14-year-old patient with pectus excavatum—postoperative result after MIRPE.

We report on a 14-year-old adolescent male who presented with a moderate asymmetric pectus excavatum (Figure 1). The patient complained about mild exercise intolerance and intermittent shortness of breath. Preoperative imaging showed a Haller index of 2.6, a correction index of 23.3, and an asymmetry index of 104.4. The patient underwent an uneventful repair of his asymmetric pectus excavatum. Bilateral thoracic cryoablation was performed, followed by placement of a single pectus bar with one stabilizer on the non-dominant side (Figure 2a). Chest X-rays obtained postoperatively and before discharge (postoperative day 3) demonstrated a small bilateral pneumothorax without significant pleural effusion (Figure 2b). The patient was discharged on postoperative day 3 in good clinical condition (Figure 3).

**Figure 4:**
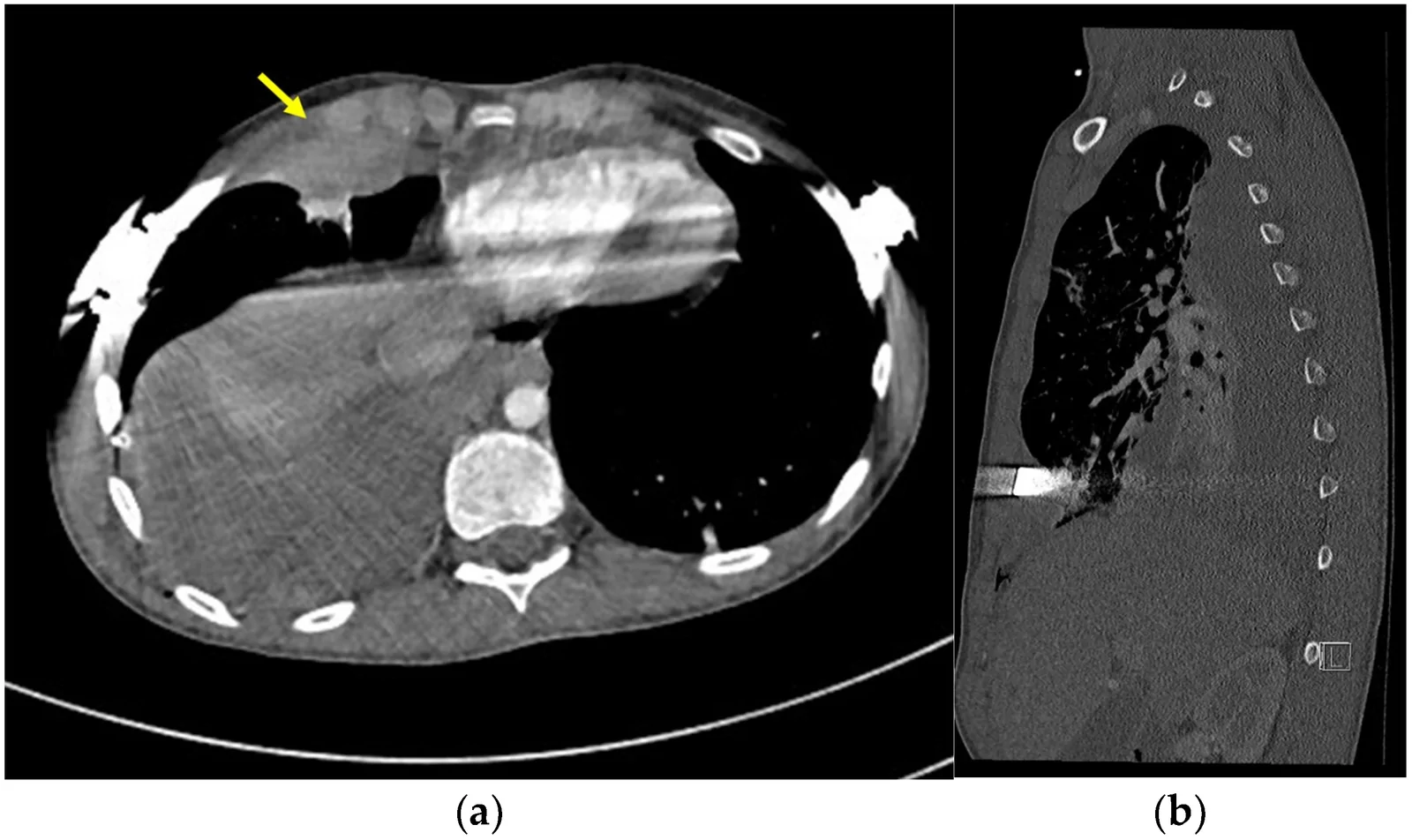
(**a**,**b**) CT angiography: suspicion of a relevant lesion to the right-sided internal mammary artery (IMA) adjacent to the pectus bar (yellow arrows).

Five weeks after MIRPE, a few days before the scheduled follow-up appointment, the patient presented to our emergency room (ER) with shortness of breath and right-sided thoracic pain. Laboratory investigations demonstrated mild leukocytosis (12.3 G/L), a normal C-reactive protein level (CRP), and a body temperature of 37.5 °C. Chest radiography demonstrated a right lower lobe opacity with a suspected positive air bronchogram, leading to a presumptive diagnosis of right lower lobe pneumonia. Treatment included antibiotic therapy with Amoxicillin (parenteral administration for 3 days, oral medication after discharge) and deep breathing exercises. Three days later, the patient returned to the ER with worsening shortness of breath, dizziness, right-sided chest pain, and vomiting. Clinical examination and chest imaging revealed a massive spontaneous right-sided hemothorax. Hemoglobin was 93 g/L on second admission, with the patient in a stable hemodynamic condition. A chest tube was inserted, with an initial drainage of 600 mL of bloody pleural effusion. CT angiography was performed to identify the bleeding source (Figure 4). Although no active contrast extravasation was observed, imaging suggested an injury to the right-sided internal mammary artery (IMA). After a total drainage of 1600 mL, the patient remained hemodynamically stable. The lowest recorded hemoglobin concentration was 93 g/L, compared with 118 g/L five days earlier. The chest tube was removed within 24 h.

Endovascular embolization of the IMA was discussed with the interventional radiology team. However, given the absence of active bleeding on CT angiography, the patient’s sustained hemodynamic stability, and the potential risks associated with embolization, conservative management was considered the most appropriate treatment strategy. Further follow-up visits showed the patient in stable clinical condition, laboratory investigations with increasing hemoglobin concentration (hemoglobin: 100 g/L 2 weeks later), and chest radiography without pleural effusion. During 32 months of follow-up, the patient remained asymptomatic. Chest radiography after 32 months demonstrated no pneumothorax, no pleural effusion, and the pectus bar (including the stabilizer) still in the correct position. Elective pectus bar removal is scheduled after another 3–4 months.

## 3. Discussion

Early case reports followed by comprehensive reviews raised awareness of the risks and complications associated with MIRPE [2,6,7,9,10]. For pectus surgeons, thorough knowledge of these risks is essential to maximize procedural safety. The introduction of routine thoracoscopy and intraoperative sternal elevation has substantially improved the safety of MIRPE and reduced the incidence of serious procedure-related complications [11,12]. However, after more than two decades after the introduction of MIRPE, the true prevalence and type of life-threatening complications related to MIRPE and subsequent PBR remain uncertain, and are likely underreported [6]. Cardiac perforation during introducer passage, pectus bar placement, or PBR represents a rare but potentially fatal complication. Likewise, accidental lesions to the internal mammary (IMA) or intercostal vessels are uncommon but may result in life-threatening hemorrhage. Previous studies have demonstrated that the pectus bars can exert local pressure on the IMA [13]. Elsayed reported compromised IMA blood flow in 44–58% of patients with the bar in situ [14], consistent with the findings of Yüksel et al., who observed impaired IMA blood flow in 44% of patients following MIRPE [13]. These observations suggest that, in addition to altered blood flow, the IMA may also be susceptible to mechanical injury or erosion. Although several studies and case reports have described accidental IMA injury during pectus repair [7], delayed (>1 month) and near-fatal hemorrhage after MIRPE has rarely been reported, and the precise source of bleeding often remains unidentified [15,16,17,18]. Adam et al. noticed erosion of the pectus bar in the IMA only after four months post-MIRPE [17].

Our 14-year-old adolescent male underwent an uneventful MIRPE and had an uneventful postoperative course across five weeks. Five to six weeks after surgery, he presented with shortness of breath and dizziness without preceding trauma or unusual physical activity. Delayed spontaneous hemothorax and/or pneumothorax, and delayed vascular bleeding after MIRPE, have been described in the literature, most commonly as isolated case reports [11,12,15,16,17,18]. In our patient, chest radiography demonstrated no secondary displacement or rotation of the pectus bar. CT angiography was therefore performed to identify the bleeding source. Although no active contrast extravasation was detected, imaging raised suspicion of injury to the right IMA. Bleeding from an intercostal vessel must be considered as another possible bleeding source. However, based on the previously reported case of IMA erosion caused by the pectus bar [17] and our careful review of the available imaging studies, we considered the right IMA to be the most likely source of bleeding after exclusion of other plausible causes. Nevertheless, we acknowledge that the bleeding source could not be confirmed definitively because CT angiography showed no active hemorrhage. Furthermore, although the initial radiographic findings were interpreted as pneumonia, retrospective review suggests that they may already have represented an early hemothorax preceding the subsequent massive hemorrhage. Closer clinical surveillance, including serial imaging over a longer observation period, might have facilitated an earlier diagnosis.

For suspected IMA injury, endovascular coil embolization by interventional radiology was discussed as one treatment option, while redo-thoracoscopy with surgical repair of the suspected lesion represented the surgical alternative. Potential side effects of endovascular coil embolization, including rare but serious neurological events such as stroke as a worst-case scenario, must be considered during decision-making. In our patient, conservative management was chosen because of sustained hemodynamic stability, the absence of active bleeding on CT angiography, spontaneous cessation of chest tube output, and continuous clinical improvement. Consequently, although the IMA was considered to be the presumed source of bleeding, this diagnosis remained indirect and was based on exclusion of other likely sources rather than definitive evidence, similar to the case reported by Adam et al. [17].

Elective PBR will be scheduled approximately three years after MIRPE, and we plan to perform PBR under direct thoracoscopic view to maximize procedural safety.

## 4. Conclusions

Pectus surgeons are well aware of the risks of cardiac perforation and/or inadvertent injury to the IMA during MIRPE. However, delayed near-fatal hemorrhage occurring more than one month after surgery should also be recognized as an extremely rare but life-threatening complication. In the present case, the precise bleeding source could not be confirmed because CT angiography demonstrated no contrast extravasation. The diagnosis of presumed IMA bleeding was therefore based on exclusion of other plausible sources in conjunction with the radiological findings. Our 14-year-old patient was successfully managed with conservative management and careful observation. Nevertheless, immediate intervention including endovascular IMA embolization or thoracoscopic surgical repair is mandatory in patients with persistent or ongoing hemorrhage. Awareness of this rare complication is important for pectus surgeons, and we encourage reporting of similar cases to improve understanding of its incidence, mechanism, and optimal management.

## Data Availability

The original contributions presented in this study are included in the article. Further inquiries can be directed to the corresponding author.
